# A Historical Review on Antibiotic Resistance of Foodborne *Campylobacter*

**DOI:** 10.3389/fmicb.2019.01509

**Published:** 2019-07-26

**Authors:** Yichao Yang, Kristina M. Feye, Zhaohao Shi, Hilary O. Pavlidis, Michael Kogut, Amanda J. Ashworth, Steven C. Ricke

**Affiliations:** ^1^ Department of Poultry Science, University of Arkansas Fayetteville, Fayetteville, AR, United States; ^2^ Southern Plains Agricultural Research Center, Agricultural Research Service, United States Department of Agriculture, College Station, TX, United States; ^3^ Center of Food Safety, Department of Food Science, University of Arkansas, Fayetteville, AR, United States; ^4^ Diamond V, Cedar Rapids, IA, United States; ^5^ Poultry Production and Product Safety Research Unit (USDA-ARS), Fayetteville, AR, United States

**Keywords:** *Campylobacter*, antibiotic resistance, macrolide, fluoroquinolone resistance, historical review

## Abstract

*Campylobacter* is one of the most commonly reported foodborne human bacterial gastrointestinal pathogens. *Campylobacter* is the etiological agent of campylobacteriosis, which is generally a self-limited illness and therefore does not require treatment. However, when patients are immunocompromised or have other co-morbidities, antimicrobial treatment may be necessary for clinical treatment of campylobacteriosis, macrolides and fluoroquinolones are the drugs of choices. However, the increase in antimicrobial resistance of *Campylobacter* to clinically important antibiotics may become insurmountable. Because of the transmission between poultry and humans, the poultry industry must now allocate resources to address the problem by reducing *Campylobacter* as well as antimicrobial use, which may reduce resistance. This review will focus on the incidence of antibiotic-resistant *Campylobacter* in poultry, the clinical consequences of this resistance, and the mechanisms of antibiotic resistance associated with *Campylobacter*.

## The Importance Of Antibiotic-Resistant *Campylobacter*

Campylobacteriosis is usually a self-limited illness, and patients with prolonged symptomology are usually placed on a macrolide or fluoroquinolone antibiotic regimen (Salazar-Lindo et al., 1986; Allos and Blaser, 2010). However, antibiotic resistance of *Campylobacter* to these important classes of antibiotics, especially fluoroquinolones, is on the rise (US Food and Drug Administration, 2014; Bolinger et al., 2018). Resistance in two important Anti*-Campylobacter* antibiotics, azithromycin (macrolide) and ciprofloxacin (fluoroquinolone), has increased, resulting in approximately 310,000 cases of potentially untreatable infections, leading to 28 deaths in the United States annually (CDC, 2019).

The rise in antibiotic resistance has been geographically heterologous, with trends in both good and bad antibiotic stewardship mirrored in the agricultural and private sector. Information learned through epidemiological studies in higher income countries is targeted toward stopping resistance. Specifically, the antibiotic stewardship programs like the National Healthcare Safety Network, Gonococcal Isolate Surveillance Program, National Tuberculosis Surveillance System, and the Emerging Infections Program have all increased awareness of the prescription behavior, patient compliance, and resistance pattern of important clinical pathogens (Ventola, 2015a,b). Many of these programs have targeted practitioners to stop over-prescribing antibiotics. Instead, data have directly resulted in a more tuned regimen program for multiple classes of clinically important antibiotics and led to improve treatment success (Fridkin et al., 2014; Ventola, 2015a,b). Specific to food, the National Antimicrobial Resistance Monitoring System was established in 1996. This program distributes information and conducts research on foodborne pathogens from retail meat and the risk may or may not pose to the community as a whole (Zhao et al., 2006; Ventola, 2015a,b). The World Health Organization has had additional resolutions to improve antibiotic stewardship as the European Union and other groups worldwide (Ventola, 2015a,b). In more developed countries, organizations track the spread of antibiotic-resistant foodborne pathogens, which in turn enable regulatory agencies to change management strategies. This awareness has brought about changes that may ultimately reduce the risk of antibiotic resistance.

While antibiotic stewardship is still a challenge in high income countries, it is not the same obstacle as what is faced in lower to middle income countries. In these countries, agriculture is exploding, and there are very few, if any, regulations on the market to control the use of antibiotics. As these countries also typically have high incidence of diarrheal disease that impacts growth and development as well as reduces vaccine efficiency, the rise in any antibiotic resistance is alarming (McCormick and Lang, 2016). In particular, countries such as Brazil, Russia, India, China, and South Africa (BRICS) require antibiotics to meet the agricultural paradigm in place (Van Boeckel et al., 2014).

In the BRICS countries, poor hygiene, lack of access to potable water, and the absolutely uncontrolled use of antibiotics all contribute to this issue of spreading disease and rise in resistance (Van Boeckel et al., 2014; Frost et al., 2019). For instance, in India, *Campylobacter* isolates are often multiple-drug resistant and have phenotypic resistance to ciprofloxacin, tetracycline, furazolidone, ampicillin, gentamicin, and erythromycin (Jain et al., 2005). In Central America, trends hold true as well, with antibiotic-resistant *Campylobacter* on the rise (Toledo et al., 2018).

Besides reducing the treatment failures for diarrheal diseases in low income countries, preventing the rise in antibiotic-resistant *Campylobacter* is essential as certain populations that are more at risk for severe complications. These populations, such as the immunocompromised or the elderly, will likely be placed on antibiotic regimens in order to prevent bacteremia and sequelae. Specifically, HIV patients are particularly vulnerable as infections can be intractable with bacteremia, ultimately requiring macrolide or fluoroquinolone therapy (Hussein et al., 2016). Another potential outcome of campylobacteriosis includes when *Campylobacter* disseminates to the periphery and causes profound disease. *Campylobacter fetus,* a rare and animal associated *Campylobacter* species epithet, causes spondylodiscitis in HIV and aged patients that commonly have other comorbidities (diabetes mellitus), where common therapeutics are not effective (Olaiya et al., 2018). Therefore, antibiotic-resistant strains of *Campylobacter* are absolutely problematic clinically as treatment resistance could have significant consequences for immunocompromised patients. As a result, understanding how resistance occurs historically is important in order to potentially find novel patterns and lessons in controlling *Campylobacter*.

A controversial avenue for the introduction of antibiotic-resistant *Campylobacter* strains to humans is through the consumption of meat, including poultry (Thakur et al., 2010; Barton, 2014; US Food and Drug Administration, 2014; Mäesaar et al., 2016). *Campylobacter* is present as a commensal organism in the gastrointestinal tracts (GITs) of poultry and can be antibiotic resistant (Zhao et al., 2010; Kojima et al., 2015; Ohishi et al., 2017; Raeisi et al., 2017). A Polish study indicated that as many as 94.4% of *Campylobacter jejuni* isolated from chicken are resistant to at least one class of antibiotic (Wieczorek et al., 2017). However, the link between agriculture and human clinical isolates of *Campylobacter* remains controversial. This review will focus on the mechanisms associated with antibiotic resistance, the controversial link between the use of antibiotics in agriculture and clinical resistance, and the current antibiotics available as well as the challenges faced with the ever-present rise in antibiotic-resistant isolates.

## Antibiotic Resistance Mechanisms

Antibiotics inhibit the growth and proliferation of microorganisms by binding to a specific target central to the microbial molecular biology and inhibiting the targets normal homeostatic activity or otherwise preventing the activity of an antibiotic. These effects can result in either bacteriostatic (cessation of replication) or bactericidal (killing) effects on the microorganism. There are four common mechanisms associated with antibiotic resistance: alteration of the antibiotic target, inactivation of the drug, decreasing membrane permeability, and the expression of antimicrobial efflux pumps (Iovine, 2013). In *Campylobacter*, mechanisms of antibiotic resistance are no different and are on occasion multimodal.

One of the most common mechanisms associated with antibiotic resistance is the ability of microorganisms to alter their membrane permeability, which ultimately prevents diffusion of the antibiotic into the intracellular environment. Porins are transmembrane proteins that create molecular pores allowing for the diffusion of chemical compounds that otherwise cannot cross the cell membrane, including antibiotics, into the periplasmic and intracellular environment (Galdiero et al., 2012). *Campylobacter* reduces membrane permeability *via* changing the expression of porins (Page et al., 1989; Pumbwe et al., 2004). In many instances, the natural and unique porins expressed by *Campylobacter* naturally prevent the entry of most antibiotics with molecular weights greater than 360 KDas (Page et al., 1989). By altering porin expression pattern, *Campylobacter* can reduce antibiotic diffusion to target within the intracellular and periplasmic space.

Another common mechanism used by *Campylobacter* for resistance is the expression of efflux pumps, which in many cases result in multidrug-resistant phenotypes. Efflux pumps can be expressed by both Gram-positive and Gram-negative prokaryotes and actively transport structurally variable molecules like antibiotics from the periplasmic or cytoplasmic space to the external environment of the cell (Pagès and Amaral, 2009; Nikaido and Pagès, 2012; Handzlik et al., 2013; Blair et al., 2014; Yao et al., 2016). An example of this includes the efflux pumps used by *Campylobacter* that removes the aminoglycosides from the intracellular space, preventing the antibiotic from reaching the ribosome and exerting its effects. Potential novel approaches to combating this kind of resistance include potentiators that act as efflux pump antagonist, which ultimately shuts down resistance (Mamelli et al., 2003; Payot et al., 2004). Efflux pumps are especially concerning as they are not specific and may successfully efflux multiple classes of antibiotics. Yao et al. (2016) found that the emergence of a super efflux pump variant, named *RE-CmeABC*, directly confers multidrug resistance in *Campylobacter* as it can non-specifically efflux multiple classes of antibiotics. *RE-CmeABC* is a particularly dangerous genetic element as it is encoded in a plasmid and subject to horizontal transfer.

Mutations in the target site reduce affinity and avidity for the antibiotic, which ultimately makes that class of antibiotics ineffective (Vetting et al., 2011). For example, fluoroquinolone resistance is due to a point mutation in the topoisomerase site where the antibiotic typically binds and renders it ineffective. Point mutations occur naturally as a consequence of normal biological replication and can be favored or disfavored by environmental conditions. By changing that target site, the point mutation reduces the binding affinity of the fluoroquinolone (Ling et al., 2003). In environments where antibiotics are non-existent, resistant point mutations are not likely a favored or even result in a neutral mutation. However, if antibiotics are present, the surviving bacterial progeny must confer that mutation for resistance and selective pressure results in the mutation becoming dominant (Ling et al., 2003). Additionally, the biotransformation of an antibiotic by the bacteria intracellularly can also render it ineffective. Bacterial enzymes in the bacteria modify side chains of chemical groups on the antibiotic, which consequentially reduce antibiotic-binding affinity to the target site. A classic example of this important mechanism occurs in aminoglycoside antibiotics, which have numerous hydroxyl and amide groups that are vulnerable to modification once in bacterial systems (Norris and Serpersu, 2013). Once modified, the aminoglycosides are ineffective.

The rise in multiple antibiotic-resistant *Campylobacter* is mechanistically multimodal. Numerous studies point to multimodal resistance becoming an important component of *Campylobacter* drug resistance. Hao et al. (2016) found that *C. jejuni* 1655 had several mutations at different target sties for antibiotics, including Thr-86-Ile mutation in *gyrA* and the A2075G mutation in *23S rRNA*, *tetO*, *aphA*, and *aadE* genes. Additionally, *C. jejuni* 1655 carried a *pTet* plasmid. All together, these mutations and plasmids result in a multidrug resistant phenotype to fluoroquinolone, macrolide, tetracycline, and aminoglycoside classes of antibiotics. Therefore, identifying one mechanism of resistance as the resistance “smoking gun” is not likely. Clinically, data indicates that *Campylobacter* should be subjected to antibiograms prior to the initiation of clinical therapeutic regimens to select antibiotics; the pathogen is susceptible in order to ensure that positive therapeutic outcomes are possible.

## Agriculture, Antibiotics, and *Campylobacter*

Significant press and public attention have, likely unfairly, pointed to the agricultural industry as the root cause of antibiotic resistance. However, this is still a controversial notion, with data resulting from multiple studies ultimately being non-conclusive. Multiple studies have been conducted to determine whether or not this link is real. Ultimately, this may be a case of correlation not indicating causation. While the rise in resistance in the clinical sector is mirrored in the agricultural sector, these may be very independent events or perhaps existing in a gray area. The judicious use of antibiotics as a whole may ultimately combat antibiotic resistance.

In order to determine if there was a link between antibiotic-resistant *Campylobacter* in poultry with human campylobacteriosis, a study was conducted that isolated *Campylobacter* strains from poultry and human clinical campylobacteriosis samples (Wieczorek et al., 2018). While some correlations existed, the link was neither direct nor concrete and remains controversial. Additionally, Silva et al. (2016) found that there is no relationship between *Campylobacter* isolated from human and poultry sources based on pulse field gel electrophoresis analysis. More research is needed to fill the gap between animal use antibiotics and human infection of antibiotic-resistant pathogens.

The paralleled rise in resistance is illustrated by the rise of quinolone-resistant strains in the veterinary and clinical setting. It is true that in the early 1990s, Endtz et al. (1991) found a rise in resistant strains isolated from poultry products from 0 to 14% between 1982 and 1989. Correspondingly, quinolone-resistant *Campylobacter* isolates increased from 0 to 11% isolated from human sources. In order to determine if agricultural isolates impacted human disease outcomes, Zhao et al. (2015) used pulsed-field gel electrophoresis (PFGE) to subtype *Campylobacter* isolates from humans and retail poultry and observed that isolates between the two sets displayed the same resistance phenotypes and PFGE patterns. Data indicated that the human isolates were likely linked to the contaminated poultry products. However, this is not entirely correlative and the exposure of humans to resistant *Campylobacter* does not come without its own nuances. Zhao et al. (2015) also observed that human isolates of *Campylobacter* tended to be more genetically diverse and resistant than the retail chicken isolates. This amplification of drug resistance in clinical patients may be due to the horizontal transfer of mobile genetic elements encoding antibiotic resistance in the human gastrointestinal tract (Barnes et al., 1972; Eckburg et al., 2005).

In order for causation to be correlative, a direct link must be established and consistent between agriculture and clinical isolates, which remains to be the case with *Campylobacter*. The missing link likely speaks to the complexities associated with mobile genetic elements making that direct link unlikely or that the correlation ultimately does not mean causation. Some models have shown that antibiotics fed to food animals present a low risk of clinical treatment failure associated with antibiotic resistance. For example, Hurd et al. (2004) conducted a risk assessment modeling the effects of tylosin and tilmicosin, two macrolide antibiotics, on *Campylobacter* spp. and *E. faecium*. The scientists administered both types of veterinary antibiotics to swine, cattle, or poultry for therapeutic, prophylactic, and growth promotion. The antimicrobial resistance determinant was considered the most likely hazard factor for causing human illness, which is supported by U.S. Food and Drug Administration’s Center of Veterinary Medicine. This farm-to-patient risk assessment model indicated that the use of macrolides resulted in less than 1 in 10 million rate of failure in treating *Campylobacter* with these two antibiotics. While resistance is a problem in both sectors, the common use of macrolides used in food animals does not impact antibiotic resistance in human campylobacteriosis.

## Antibiotics Used to Treat Campylobacteriosis in the Clinical Setting

The CDC has recommended treatment regimens be initiated for *Campylobacter* if the patients have weakened immune systems or are experiencing profound effects. Commonly, macrolides and fluoroquinolones are prescribed with macrolides being preferred due to their low resistance rate (CDC, 2019). It has been documented that there are a greater frequency in identifying fluoroquinolone-resistant isolates compared to macrolide-resistant *Campylobacter* isolates. That may be due to the mutation frequency of the 50S ribosomal subunit that confers resistance occurring in approximately 10 mutations per *Campylobacter* cell per generation. The mutation frequency for the 50S ribosomal subunit is 10,000-fold lower than the mutation frequency of the gyrase and topoisomerase genes that confer fluoroquinolone resistance (Yan et al., 2006; Lin et al., 2007). The increased rate in mutation frequency likely corresponds to the rise in ciprofloxacin-resistant isolates *Campylobacter,* which was from 13% in 1997 to 19% in 2001 (Gupta et al., 2004). This is compared to the steady prevalence of 2% erythromycin, a macrolide, resistant positive isolates of *Campylobacter* during the same time period (Gupta et al., 2004). This trend has continued through time as currently the prevalence of fluoroquinolone resistance is 35.4% in *C. jejuni* and 74.4% in *Campylobacter coli* in the United States (Tang et al., 2017).

## Macrolides

A course of the macrolide azithromycin is the gold standard chemotherapeutic regimen to treat *Campylobacter* infections, which effectively reduces bacterial shedding and the length of the illness (Kuschner et al., 1995). The breakpoint concentration associated with azithromycin indicates that 30 to 500/d for 3 days of azithromycin was effective for the eradication of *Campylobacter* and acceleration of a patient’s recovery time (Kuschner et al., 1995; Vukelic et al., 2010). This, as well as the low incidence of natural resistance, has led to azithromycin being the drug of choice (DuPont, 2007).

Another option for therapeutic regimens in clinical medicine includes the use of erythromycin (Guerrant et al., 2001). Efficacy is lost when the antibiotic regimen is not started at the onset of symptoms and may require prolonged treatment (Guerrant et al., 2001). Advantages of using erythromycin include the low frequency of natural resistance of *Campylobacter* to erythromycin (Smith et al., 1999; Bardon et al., 2009), with antibiogram analysis of 1,808 isolates from Finnish patients between 2003 and 2005 reporting a resistance prevalence to erythromycin at 1.1% (Lehtopolku et al., 2010). Clindamycin is a lincomycin antibiotic, and Wagner et al. (2003) first proposed this as a potential therapeutic for campylobacteriosis. This alternative was evaluated by determining the minimum inhibitory concentration for five common antibiotics. The researchers observed that only 2% of the *Campylobacter* isolates were resistant to clindamycin, while greater than 45% of isolates exhibited resistance against four fluoroquinolone antibiotics tested (Wagner et al., 2003).

## Fluoroquinolones

Fluoroquinolones may be used to treat *Campylobacter* but are problematic due to the resistance profile of clinical *Campylobacter* isolates (Nord and Edlund, 1991). The clinical isolates associated with active campylobacteriosis exhibit greater frequencies of quinolone resistance, increasing as much as 20-fold in the 1990s in Sweden (Wretlind et al., 1992; Gibreel et al., 1998). As with macrolide resistance, fluoroquinolone resistance is naturally occurring as a point mutation, ultimately leading to treatment failure and symptomatic relapse (Segreti et al., 1992; Sanders et al., 2007). A classic example of this issue is norfloxacin, fluoroquinolone widely used to treat enteric infections (Sjögren et al., 1997). Epidemiological evidence indicates that resistance can occur within 1 day of therapy (Sjögren et al., 1997). In fact, resistance to multiple fluoroquinolones exhibits a sharp increase in *Campylobacter* isolates, such as the rise of nalidixic acid resistance from 8.2% in 1990 to 26.3% in 2004 (Gallay et al., 2007), and a similar trend of quinolone resistance occurred in Germany (Luber et al., 2003).

There are promising fluoroquinolones for use in the clinical setting, yet mounting resistance continues to be a significant theme in the historical perspective associated with *Campylobacter* fluoroquinolone resistance. For instance, moxifloxacin is a fourth-generation synthetic fluoroquinolone that effectively kills *Campylobacter* and is difficult to mount resistance toward, as compared to ciprofloxacin (Wagner et al., 2003). Additionally, the use of levofloxacin in clinical cases of campylobacteriosis is promising (de la Cabada Bauche and DuPont, 2011). The effectiveness of a single dose treatment of 500 mg/d of levofloxacin is similar to 1,000 mg/d of azithromycin but with fewer side effects (Sanders et al., 2007). However, as with most fluoroquinolones, resistance has been observed when compared with azithromycin (Sanders et al., 2007).

Resistance against other fluoroquinolones has been widespread. A common fluoroquinolone of choice for medical practitioners, ciprofloxacin, has been reported in developing countries with levels ranging from 30 to greater than 84% (Hoge et al., 1998; Pandey et al., 2010; Meng et al., 2011). As with other fluoroquinolones, the frequency of isolating ciprofloxacin-resistant strains of *Campylobacter* is rising. In Peru, Pollett et al. (2012) observed that between 2001 and 2010, the prevalence of ciprofloxacin-resistant *Campylobacter* isolates rose from 73.1 to 89.9% in the area of Lima and from 24.1 to 48.9% in the Iquitos region. Similar to the clinical setting, an increased prevalence of fluoroquinolone-resistant *Campylobacter* strains has been recovered from food animals in developed countries (Taylor et al., 2008). Nannapaneni et al. (2005) isolated ciprofloxacin-resistant *Campylobacter* isolates from retail raw chicken carcasses in the United States with numbers ranging from 57% in 2001 to 96% in 2003. This is likely due to the relationship developing countries have with antibiotics, which ultimately results in poor antibiotic stewardship and an ultimately significant increase in antibiotic resistance. Therefore, increase in both clinical and veterinary isolates of *Campylobacter* in the developing world is anecdotal of a larger problem in countries where the use of antibiotics in both independent settings, the clinical and the veterinary, is poorly controlled and executed.

## Models for Controlling *Campylobacter*

The intensive use of antibiotics has been suggested to have an increased number of resistant strains of mutants and a decreased effectiveness of antibiotics (Wegener, 2003; Ventola, 2015a,b). The World Health Organization (WHO) has concluded that increases in antibiotic resistance represent a considerable, worldwide threat to public health (WHO, 2014). In order to attempt to preserve the efficacy of clinical antibiotics, the European Union banned the use of clinical antibiotics for growth promotion in 1999 (Casewell et al., 2003). In the United States, the Institute of Medicine recommend to reduce or eliminate the use of antibiotics in feed in 1980 and 1989, and also it was supported by a Council for Agricultural Science and Technology (1981) and a Committee on Drug Use in Food Animals Panel on Animal Health, Food Safety, and Public Health (1998). The Veterinary Feed Directive was recently implemented in the United States to limit the use of human clinical antibiotics as well as provide stricter guidelines for the use of antibiotics in food animals and is viewed as the first step to solving a significant veterinary and human clinical problem (Veterinary Feed Directive (VFD), 2017).

However, despite the regulations, it is still under debate about the relationship between resistance microorganisms selected when an antibiotic was used as the growth promoter in food animals and the antibiotic-resistant infections in humans (Phillips et al., 2004; Vaughn and Copeland, 2004). At the time of increasing concern about the spread of antibiotic resistance pathogens in humans, U.S. Food and Drug Administration initiated the Veterinary Feed Directive (VFD), which requires a prescription from a veterinarian to use medically important antibiotics in infected animals. These medically important antibiotics are not allowed to be used as growth promoters. The regulation information has been listed in Table 1.

**Table 1:** Veterinary feed directive antibiotics.

	Medically important drugs	Non-medically important drugs
Therapeutic uses	Allow under veterinary supervision	Allow under veterinary supervision
Production use	No longer allowed	Allowed
Drugs	Penicillin Cephalosporins Quinolones Fluoroquinolones Tetracyclines Macrolides Sulfas Glycopeptides	Bambermycin Carbadox Ionophores Pleuromutilin Polypeptides

As with other bacteria, the use of the correct antibiotic with the correct species epithet is important and is a tenant of good antimicrobial stewardship. When analyzing antibiotic-resistant isolates of *Campylobacter* isolated from live broilers, Li et al. (2017) reported that *C. coli* exhibited significantly greater prevalence of antibiotic resistance than *C. jejuni* to clindamycin, gentamicin, and kanamycin, but less resistance to florfenicol. This finding demonstrated that while a particular antimicrobial may be effective for reducing *C. jejuni*, it may not be as effective for treating *C. coli* and vice versa. Therefore, while campylobacteriosis tends to be binned within a single category, the identification of the specific species epithet is important for treatment success. By becoming more aware of the potential differences in resistance across species epithets, properly prescribing the correct treatment regimen may further enhance antimicrobial stewardship.

## Conclusion

Ultimately, it is poorly understood as to how fluid the *Campylobacter* genome is and the full risk associated with antibiotic resistance in the agricultural setting. Additionally, the link between agricultural antimicrobial abuse and treatment failures in the clinical setting still fails to be established. With increased antibiotic vigilance, research into the mechanisms that drive antibiotic resistance in *Campylobacter*, and the potential development of novel antimicrobial strategies, it may be possible to mitigate the effect of antibiotic resistance independently across the veterinary and clinical sectors. While resistance is not conferred between poultry and the clinical patient, the exposure to the clinical patient does occur through poultry. The rearrangement and transference of mobile genetic elements within the human host, the environment, and the poultry itself likely make the “smoking gun” improbable.

However, the establishment and prescription of a single-hurdle approach to control this foodborne pathogen and ultimately reduce the risk to the human population are unlikely. Therefore, not only does research need to focus on the development of new antibiotics to help patients in the clinical setting, it needs to focus on preventing the reservoir for campylobacteriosis, poultry, from continuing to serve as such. Whether or not reducing *Campylobacter* resistance in poultry affects the war on antibiotic resistance is unknown and will remain a mystery.

## Author Contributions

YY, KF, SR, AA, MK, and HP provided the framework and concept for the publication. YY produced the first version of the manuscript. KF completed, edited, and restructured the manuscript for publication. KF submitted the manuscript and handled all communications with the reviewers. All authors reviewed the manuscript prior to submission.

### Conflict of Interest Statement

HP is an employee of Diamond V. The remaining authors declare that the research was conducted in the absence of any commercial or financial relationships that could be construed as a potential conflict of interest.
